# Voltammetric determination of polyphenolic content in pomegranate juice using a poly(gallic acid)/multiwalled carbon nanotube modified electrode

**DOI:** 10.3762/bjnano.7.103

**Published:** 2016-07-29

**Authors:** Refat Abdel-Hamid, Emad F Newair

**Affiliations:** 1 Unit of Electrochemistry Applications (UEA), Chemistry Department, Faculty of Science, Sohag University, Sohag 82524, Egypt

**Keywords:** electrochemical sensor, gallic acid, multiwalled carbon nanotubes, pomegranate juice, total phenolic content

## Abstract

A simple and sensitive poly(gallic acid)/multiwalled carbon nanotube modified glassy carbon electrode (PGA/MWCNT/GCE) electrochemical sensor was prepared for direct determination of the total phenolic content (TPC) as gallic acid equivalent. The GCE working electrode was electrochemically modified and characterized using scanning electron microscope (SEM), cyclic voltammetry (CV), chronoamperometry and chronocoulometry. It was found that gallic acid (GA) exhibits a superior electrochemical response on the PGA/MWCNT/GCE sensor in comparison with bare GCE. The results reveal that a PGA/MWCNT/GCE sensor can remarkably enhance the electro-oxidation signal of GA as well as shift the peak potentials towards less positive potential values. The dependence of peak current on accumulation potential, accumulation time and pH were investigated by square-wave voltammetry (SWV) to optimize the experimental conditions for the determination of GA. Using the optimized conditions, the sensor responded linearly to a GA concentration throughout the range of 4.97 × 10^−6^ to 3.38 × 10^−5^ M with a detection limit of 3.22 × 10^−6^ M (S/N = 3). The fabricated sensor shows good selectivity, stability, repeatability and (101%) recovery. The sensor was successfully utilized for the determination of total phenolic content in fresh pomegranate juice without interference of ascorbic acid, fructose, potassium nitrate and barbituric acid. The obtained data were compared with the standard Folin–Ciocalteu spectrophotometric results.

## Introduction

Gallic acid (GA) is a natural polyphenolic compound found in fruits, vegetables and several other plants [1]. The study of the role of GA in providing better therapeutic outcomes against arsenic-induced toxicity showed that GA is effective against arsenic-induced oxidative stress [2]. A facile and ultrasensitive sensor based on gold microclusters electrodeposited on sulfonate-functionalized graphene that was immobilized on the surface of a GCE was fabricated and applied for the simultaneous determination of gallic acid and uric acid [3]. The electrochemical mechanism and optimal test conditions of GA were carefully investigated on a sensor based on chitosan/fFe_2_O_3_/reduced graphene oxide/GCE. Under optimal conditions, the detection limit was estimated to be 1.5 × 10^−7^ M [4]. An electrochemical sensor coupled with an effective flow-injection amperometric system was developed for determination of GA in a mild neutral conditions. The sensor is based on a poly (melamine) film immobilized on a preanodized screen-printed carbon electrode [5]. A voltammetric determination of GA on a hanging mercury drop electrode was investigated by cathodic adsorptive stripping voltammetry [6]. A sensitive and reliable method was developed using a differential pulse polarographic method for determination of GA in fruit juices with a detection limit of 0.3 µM [7]. Electrochemical sensors based on a carbon paste electrode modified with SiO_2_ nanoparticles [8] and carbon nanotubes [9–10] were utilized for determination of gallic acid. A sensor based on a carbon paste electrode modified with multiwalled carbon nanotubes was used for voltammetric determination of ellagic acid and gallic acid in an *Myrtus communis*, *Punica granatum* and Itriphal formulation [10]. Glassy carbon electrodes modified with a multiwalled carbon nanotube/*o*-dianisidine derivative [11] and a molecularly imprinted polypyrrole polymer-based film [12] were used for gallic acid analysis with high selectivity. A bimediator amperometric sensor for gallic acid was fabricated by surface modification of a graphite electrode with thionine and nickel hexacyanoferrate [13]. A polyethyleneimine-functionalized graphene oxide modified glassy carbon electrode sensor was developed for sensitive detection of gallic acid [14]. A polyepinephrine modified glassy carbon electrode electrochemical sensor was developed for adsorptive stripping voltammetric determination of gallic acid and successfully applied for the estimation of GA in black tea [15]. The determination of gallic acid and caffeic acid was conducted by using a stable sensor based on a Zn–Al–NO_3_ layered double hydroxide film/glassy carbon electrode [16].

Recently, pomegranate juice has attracted more scientific attention because of its valuable health effects; especially due to its high content of phenolic compounds [17]. It is a complex drink and a rich source of antioxidants such as phenolic acids, tannins, anthocyanins, procyanidins, and flavonol glycosides [18]. Due to the positive effects of polyphenol antioxidants on human health, the improvement of sensitive and robust methods for their determination gains more importance. Consequently, the measurement of the total polyphenolic content (TPC) is a good representation of the level of antioxidants that exist in a sample [19–21].

The selective and sensitive determination of TPC (GA equivalent, mg GAE L^−1^) is a difficult task, thus simple and fast techniques are still needed for these purposes. Therefore, in continuation of our previous work on the electrochemical study of antioxidants [15,22–27], the objective of present study is to develop a sensitive electrochemical method for the determination of the total phenolic content using poly(gallic acid)/multiwalled carbon nanotube/glassy carbon electrode (PGA/MWCNT/GCE) electrochemical sensor. The polymer may overcome the slow mass transfer on bare GCE or MWCNT/GCE. Thus the modification enhances the redox peak current and could be used with high sensitivity. Recently, the use of nanomaterials for electrode modification has grown exponentially, owing to their advantageous electrochemical properties. Carbon nanotubes are the main representatives of nanomaterials used in the construction of electrochemical sensors with good performance. Multiwalled carbon nanotubes (MWCNTs) were selected due to their advantages such as rapid electron transfer rate and high electrocatalytic activity. The glassy carbon working electrode was electrochemically modified and characterized using scanning electron microscope (SEM), cyclic voltammetry, chronoamperometry and chronocoulometry. To validate the suggested procedure, the determination of total phenolic content in pomegranate juice was performed using square-wave voltammetry. The results collected at optimal conditions were compared with the standard Folin–Ciocalteu spectrophotometric data.

## Results and Discussion

### SEM characterization of PGA/MWCNT composite film

**Figure 1:**
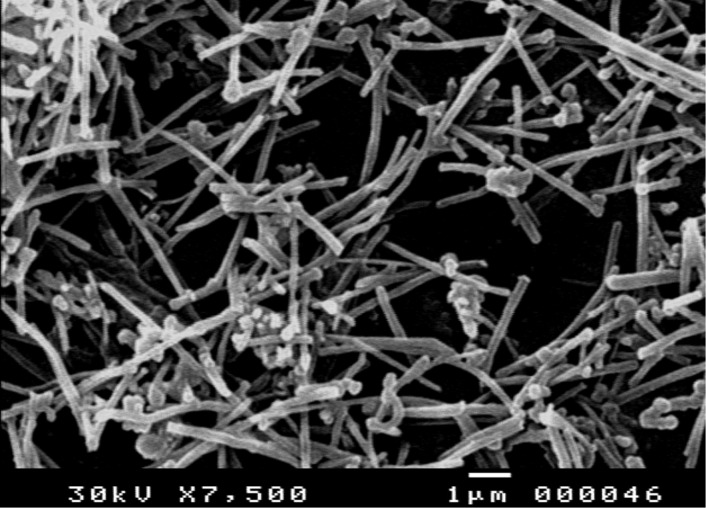
SEM morphology of a PGA/MWCNT film nanocomposite at 7,500 magnification.

The response of the prepared electrochemical sensor is related to its physical morphology. The surface morphology of the PGA/MWCNT film on a rectangular indium tin oxide (ITO) coated glass slide (resistivity of 8–12 *Ω*/□) was examined using SEM (Figure 1). The film is deposited on the ITO with the same experimental conditions as for the GCE. As shown, the nanocomposite has a short, tubular topology with a smooth surface. The nanocomposites are uniformly distributed and held together into bundles. The white circular species represent the ends of the CNTs on the surface.

### Electrochemical characterization of PGA/MWCNT/GC modified electrode

#### Cyclic voltammetry

**Figure 2:**
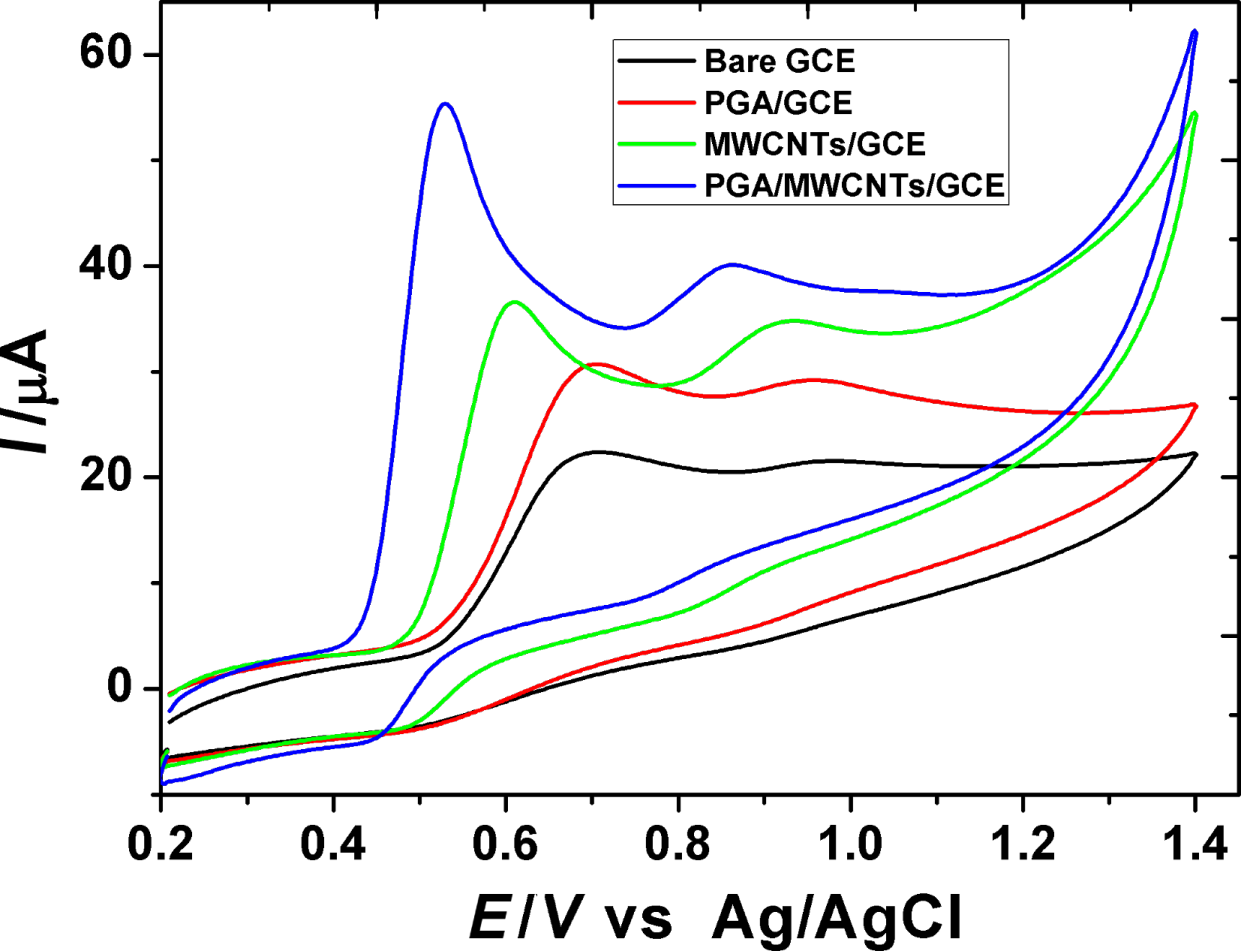
Cyclic voltammograms of 1.0 mM GA in 0.2 M H_3_PO_4_ at a scan rate of 50 mV s^−1^ on bare GCE, PGA/GCE, MWCNT/GCE and PGA/MWCNT/GCE.

Cyclic voltammograms of 1.0 mM gallic acid in 0.2 M H_3_PO_4_ at three different glassy carbon modified electrodes, PGA/GCE, MWCNT/GCE and PGA/MWCNT/GCE, were recorded at a scan rate of 50 mV/s (Figure 2). Gallic acid shows two irreversible cyclic voltammetric waves on anodic potential sweeping, lacking the corresponding reduction counterparts. An increase in the peak current and a shift of the peak potentials towards less positive potential values are observed upon the electrochemical modification of the GCE. This reveals that the electrode kinetics is improved. Upon examination of the voltammetric data, it is observed that the first anodic wave is highly sensitive to the modification. GA shows the first CV wave with a peak potential (*E*_p_^a^) at 0.69 V on the bare GCE. The *E*_p_^a^ is shifted to 0.61 and 0.53 V on MWCNT/GCE and PGA/MWCNT/GCE, respectively. Furthermore, a significant enhancement in peak current is observed on the two modified electrodes. The enhancement on MWCNT/GCE and PGA/MWCNT/GCE is 1.94 and 2.94 times, respectively, relative to the bare GCE. These observations reveal that the modification of the GCE shows a significant effect on the electrochemical oxidation response of GA. Thus, one can conclude that a PGA/MWCNTS/GCE sensor provides higher activity towards GA oxidation, improves the electrode kinetics, and decreases its over potential of oxidation.

The cyclic voltammetric behavior of K_3_[Fe(CN)_6_] on a bare GCE and PGA/MWCNT/GCE modified electrodes was investigated for the determination of true electroactive surface area (data not shown). The area can be estimated for a reversible and diffusion-controlled process according to the Randles–Sevcik [28] equation,

[1]
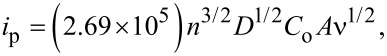


where *i*_p_ is the peak current, *n* is the number of electron transfers in the reaction (which is equal to 1), *D* is the molecular diffusion coefficient (cm^2^/s) in solution, *A* is the active surface area (cm^2^), *C*_o_ is the concentration (mol/cm^3^) of the probe molecule in the solution, and ν is the scan rate (V/s). The electroactive surface area (*A*) of the bare glassy electrode and its modified composite electrode was determined by cyclic voltammetric response using 1.0 mM solution of K_3_[Fe(CN)_6_] in 0.2 M potassium chloride solution at a scan rate of 50 mV/s. It was known that the electrochemical reduction of the ferricyanide ion at the GCE is diffusion-controlled. From Equation 1, the electroactive surface area of the subject electrodes was evaluated taking into account a diffusion coefficient for ferricyanide ion of 7.6 × 10^–6^ cm^2^/s in 0.2 M KCl [29]. The estimated active surface area values are 0.050 and 0.077 cm^2^ for bare GCE and PGA/MWCNT/GCE, respectively.

#### Chronoamperometry

**Figure 3:**
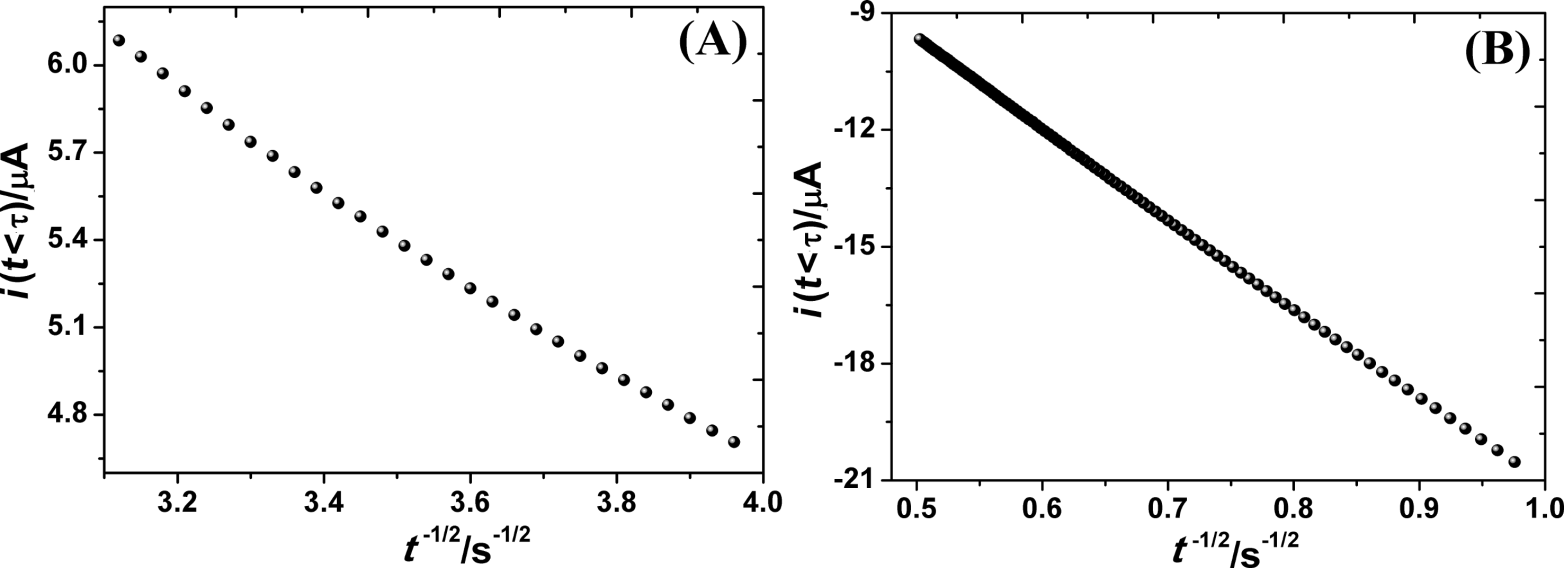
Relationship of *i*(*t* < τ) vs (*t*^−1/2^) chronoamperometry of 1.0 mM K_3_[Fe(CN)_6_] in 0.2 M KCl on (A) GCE and (B) PGA/MWCNT/GCE.

For comparison, chronoamperometric measurements were employed for estimation of the electroactive surface area. The chronoamperometric behavior of 1.0 mM K_3_[Fe(CN)_6_] on GCE and PGA/MWCNT/GCE in 0.2 M KCl solution for the first wave at different duration times was performed. For chronoamperometric experiments, the electrode potential was stepped from 0.50 to 0.02 V on GCE and from 0.050 to −0.20 V on PGA/MWCNT/GCE for a fixed duration, τ. The current that passes during τ is measured. The current corresponding to the electrochemical reaction is described by Cottrell's equation [28]:

[2]
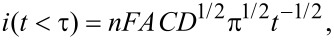


where *D* is the diffusion coefficient (cm^2^/s), *C* is the bulk concentration (mol/dm^3^), τ is the step duration and *n*, *F*, and *A* have their usual significance. According Cottrell's equation, upon plotting the current response against *t*^−1/2^, a straight linear line is obtained with a correlation coefficient of 0.999 for all duration times (Figure 3). From the slope, the active surface area was determined. The area of the working electrodes was found to be 0.051 and 0.072 cm^2^ for bare GCE and PGA/MWCNT/GCE, respectively. These values are close to the values obtained using the voltammetric method.

**Figure 4:**
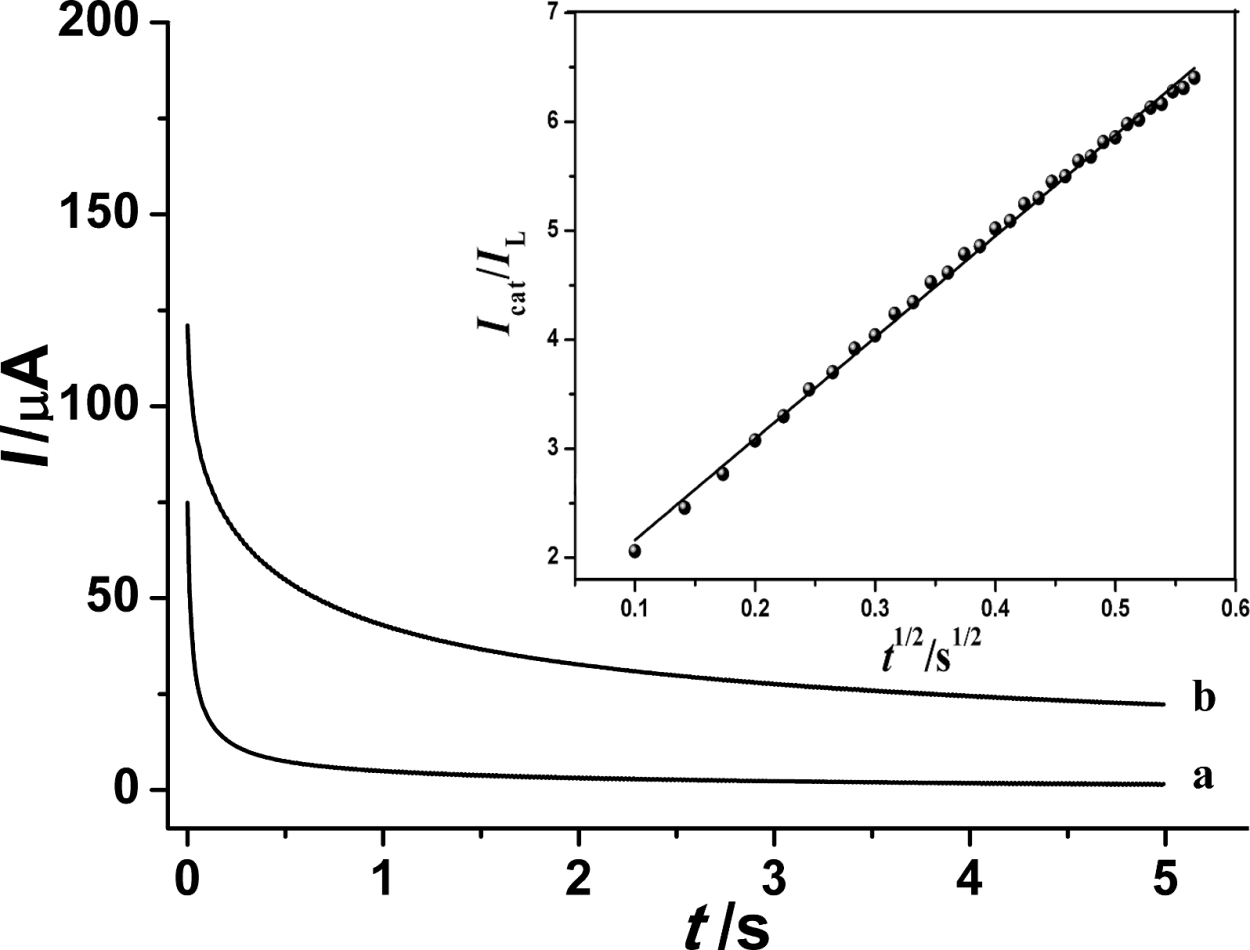
Chronoamperograms of PGA/MWCNT/GCE in 0.2 M phosphoric acid in absence (curve a) and presence (curve b) of 1.0 mM GA; the inset shows the relationship of *I*_cat_/*I*_L_ versus *t*^1/2^.

The catalytic rate constant (*k*_cat_) can be evaluated using chronoamperometry. It was determined upon performing chronoamperometry on 0.2 M H_3_PO_4_ solutions in absence and presence of gallic acid. Figure 4 shows the obtained chronoamperograms of GA on the PGA/MWCNT/GCE. The *k*_cat_ value for the oxidation reaction between GA and the modified GCE was determined using the following equation [30]:

[3]
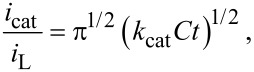


where *i*_cat_ and *i*_L_ are the currents at constant time in presence and absence of GA, respectively; *k*_cat_ is the catalytic rate constant (mol L^−1^ s^−1^), *C* is the bulk concentration of GA (M) and *t* is the elapsed time (s). The value of *k*_cat_ was estimated from the slope of the *i*_cat_/*i*_L_ ratio versus *t*^1/2^ relationship (inset of Figure 4). At 1.0 mM GA, the determined catalytic rate constant value was found to be 2.75 × 10^4^ mol L^−1^ s^−1^.

### Electrochemical oxidation behavior of gallic acid on PGA/MWCNT/GC modified electrode

#### Effect of scan rate

**Figure 5:**
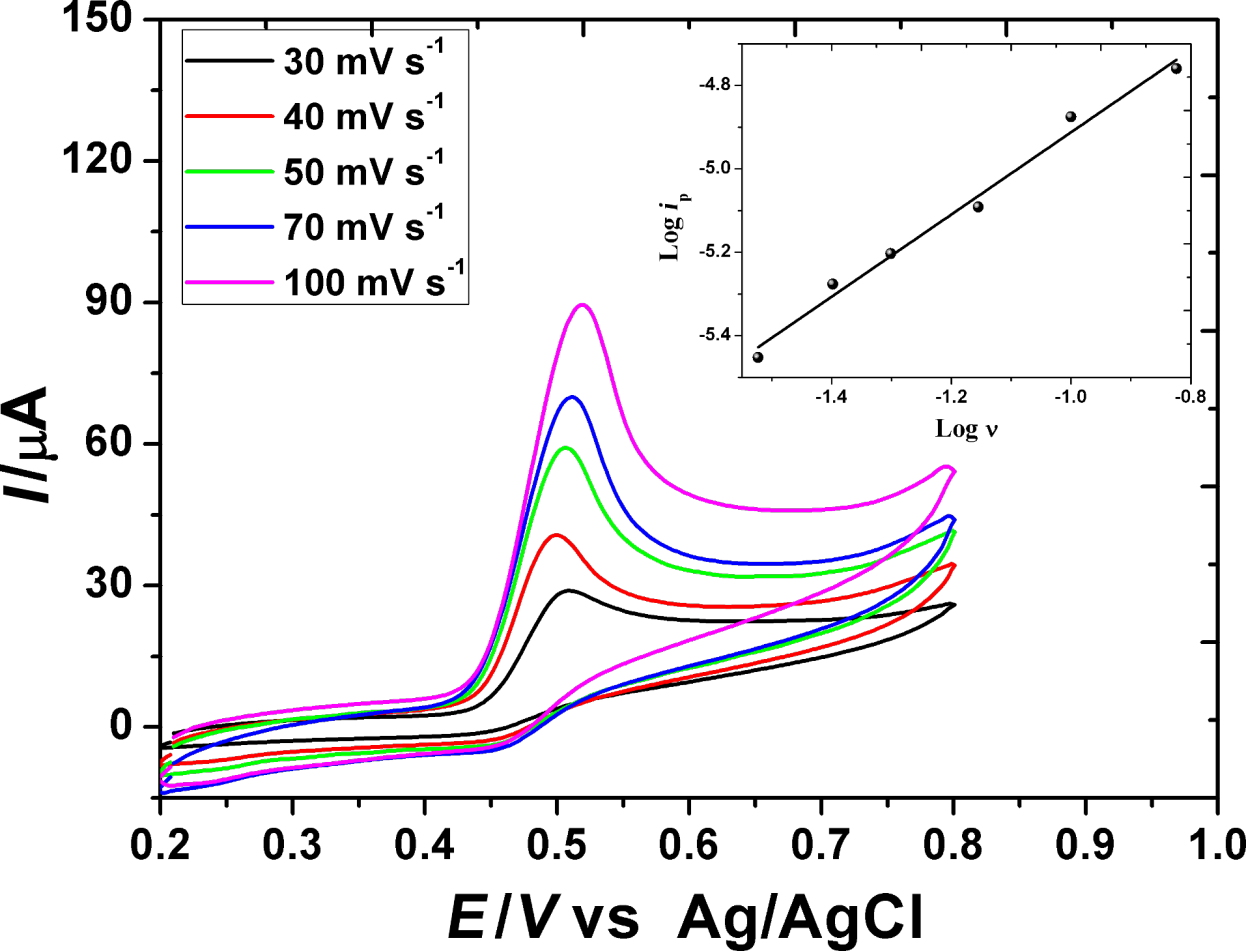
Effect of scan rate on the cyclic voltammograms recorded for the first wave of 1.0 mM GA on the PGA/MWCNT/GCE modified electrode in 0.2 M H_3_PO_4_ (pH 2.0). Inset: log *i*_p_^a^–log 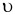 relationship.

The cyclic voltammetric behavior of 1.0 mM GA was recorded on PGA/MWCNT/GCE in 0.2 M H_3_PO_4_ (pH 2.0) at a scan rate of 50 mV/s. Gallic acid shows two irreversible cyclic voltammetric waves. This behavior was reported earlier by Abdel-Hamid and Newair [27]. The first anodic CV wave was attributed to one-electron transfer oxidation of the −OH group to form an *o-*semiquinone radical cation, which is neutralized on deprotonation. The neutral radical is further oxidized by irreversible loss of the second electron transfer and second proton to the final product giving the second anodic voltammetric wave. The effect of scan rate on cyclic voltammograms of GA was studied on the PGA/MWCNT/GC modified electrode (Figure 5). Upon increasing the scan rate (ν) in the potential scan rate range of 10–100 mV/s, the peak current (*i*_p_^a^) is proportionally increased with ν. The relationship between the oxidation peak current (log *i*_p_^a^) and the scan rate (log ν) was constructed (inset of Figure 5). It was found that the log–log plot has a straight linear relationship with a correlation coefficient of 0.972. The linear least-square relationship is represented as

[4]
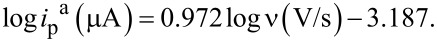


This confirms that the oxidation process is adsorption-controlled process. It concluded that the PGA/MWCNT film facilitates the electron transfer and adsorption of GA onto the electrode surface.

#### Accumulation conditions

The square wave adsorptive stripping voltammetric (SWAdSV) method was used for the electrochemical determination of GA using the prepared PGA/MWCNT/GCE sensor. The optimum conditions, accumulation potential, accumulation time and pH were tested using 0.2 M H_3_PO_4_ containing 1.0 mM GA. They were performed by measuring the peak current (*i*_p_^a^) upon varying each parameter. The effect of accumulation potential on the oxidation peak current of GA at pH 2.0 and an accumulation time of 60 s at different accumulation potentials was carried out. Upon increasing the potential from 0 V, the *i*_p_^a^ gradually increases and reaches a maximum value at a potential of +0.4 V. Upon further increase of potential, a decrease of the *i*_p_^a^ was observed. Thus, the optimal accumulation potential of +0.4 V was chosen for the subsequent experiments. The effect of accumulation time on the oxidation peak current at pH 2.0 and accumulation potential of +0.4 V was performed at different times. It was observed that the *i*_p_^a^ is increased with increasing time from 30 s to a maximum value at 60 s. Upon a further increase of time, a decrease in *i*_p_^a^ is observed. Therefore, a time of accumulation of 60 s was applied in the subsequent experiments. To optimize the solution pH for the electrocatalytic response of the PGA/MWCNT/GCE towards GA oxidation, at an accumulation potential of +0.4 V and an accumulation time of 60 s, the effect of pH was studied in the pH range of 2.3–5.5. It was observed that the anodic peak potential does shift negatively with increasing pH value. This indicates that a deprotonation reaction took part in the GA oxidation reactions. Upon increasing the pH of the solution from pH 2.3, the oxidation peak current increases to a maximum value at pH 2.6, and then it decreases upon further increase in solution pH. This indicates that the highest oxidation current is obtained at pH 2.6. Thus, it is concluded that the optimal conditions of accumulation potential +0.4 V, accumulation time 60 s and pH 2.6 will be used for GA determination with the PGA/MWCNT/GCE.

#### Calibration curve, detection limit, interference, repeatability and stability

**Figure 6:**
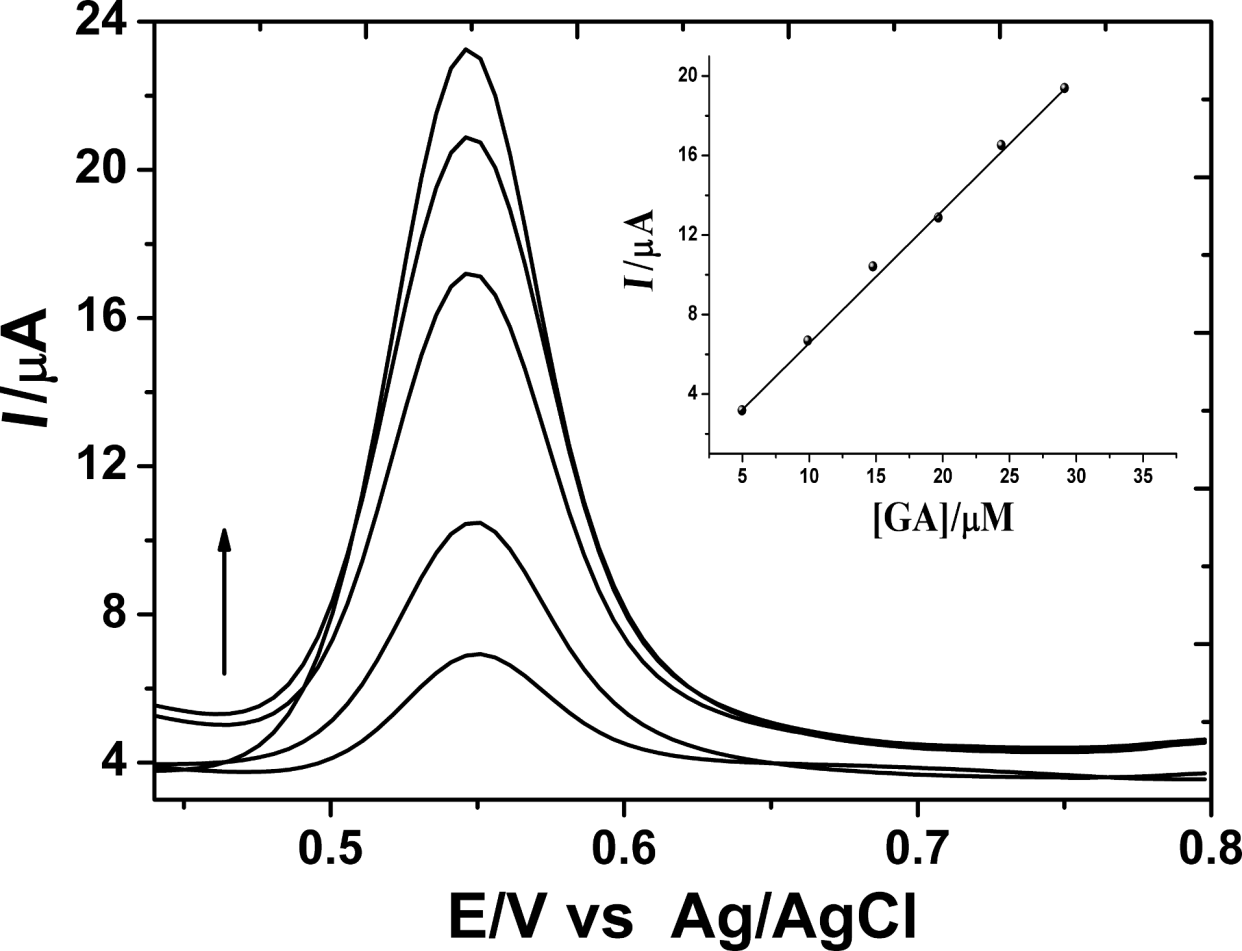
SW voltammograms obtained at optimal conditions in 0.2 M H_3_PO_4_ solution containing different GA concentrations. Inset: analytical curve.

It is well known that the square wave adsorptive stripping voltammetric method (SWAdSV) is an effective and rapid electroanalytical method with well-established advantages, including good discrimination against background and low yield detection limits. After optimization of the accumulation conditions, the sensitivity and lower detection limit tests for GA on the PGA/MWCNT/GC electrode in 0.2 M H_3_PO_4_ solution (pH 2.6) were performed. The SW voltammogram profiles of different GA concentrations are shown in Figure 6. A well-defined and sharp oxidation peak is observed on successive additions of standard solution of GA. On plotting the peak current versus GA concentration, the corresponding calibration plot was obtained (inset of Figure 6). It is found that the peak current of GA correlates linearly with the GA concentration in the range of 4.975 × 10^−6^ to 3.381 × 10^−5^ M with a limit of detection (LOD) of 3.22 × 10^−6^ M. The regression relationship is expressed as

[5]
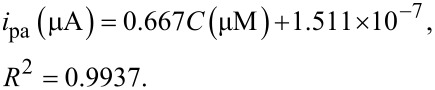


The lower limit of detection was calculated from the calibration data using the following equation:

[6]
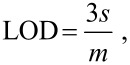


where *s* is the standard deviation of the intercept and *m* is the slope of the regression line. The analytical performance of the prepared sensor is compared with that of other GA determination methods reported previously as shown in Table 1. The estimated LOD value herein is more or less similar to those previously published using other electroanalytical methodologies. It clear that the present method is fast due to elimination of the extraction and preconcentration steps of the analyte necessary for chromatographic techniques. Furthermore, the present accumulation time is shorter than the other methods with preconcentration time of 15 min, while in the present study the optimal accumulation time was 60 s.

**Table 1:** Comparative results of GA detection on various electrodes.

electrode	linear range (µM)	LOD (nM)	ref.

AuMCs/SF-GR/GCE^a^	8.0–500.0	10.70	[3]
CS/fFe_2_O_3_/ERGO/GCE^b^	10.0–100.0	150.00	[4]
SPCE/PME^c^	0.5–500.0	76.00	[5]
DME	1.0–50.0	300.00	[7]
SiO_2_/CPE^d^	0.8–100.0	250.00	[8]
CNT/CPE	0.5–15.0	300.00	[9]

^a^Gold microclusters/sulfonate functionalized grapheme/GCE; ^b^Chitosan/fishbone-shaped Fe_2_O_3_/reduced graphene oxide/GCE; ^c^Pre-anodized screen-printed carbon electrode/polymelamine; ^d^Nano-SiO_2_/carbon paste electrode.

Selectivity determination is always a consideration in electroanalytical techniques. Therefore, the response of the SWAdSV method for GA on PGA/MWCNT/GCE in 0.2 M H_3_PO_4_ with some possible interfering compounds was studied. It is known that ascorbic acid (AA) is one of the main components present in natural samples. A fixed amount of GA was taken with different amounts of AA. On increasing the concentration of AA to a 1000-fold excess, no influence on the GA response was observed. Moreover, interference from fructose, potassium nitrate and barbituric acid was studied. It was revealed that the response of GA exhibits no change on increasing concentration of these compounds up to a 100-fold excess. This indicates that the PGA/MWCNT/GCE shows higher selectivity for GA. The precision and accuracy of the subject sensor were evaluated by examining the reproducibility and repeatability for many experimental trials. The reproducibility for GA was determined by measuring the relative standard (RSD) value of the oxidation peak current at a fixed concentration of 1.25 × 10^−6^ M GA. The RSD value obtained for the GA response was 2.45%. The repeatability of the modified electrode was evaluated with the same GA concentration. For five successive measurements, the response for the same solution containing 1.45 × 10^−6^ M GA was found to be 2.85%. These results indicate that the method provides a suitable repeatability and reproducibility in the analytical determination of GA. The recovery value of the method was determined to be 101%. Furthermore, the modified electrode exhibits good stability where as much as 95% of the initial peak current was preserved after storage for 3 weeks. This suggests that the efficiency of the PGA/MWCNT/GCE electrode for determination of GA is suitable for practical applications.

#### Determination of total phenolic content in pomegranate juice

**Figure 7:**
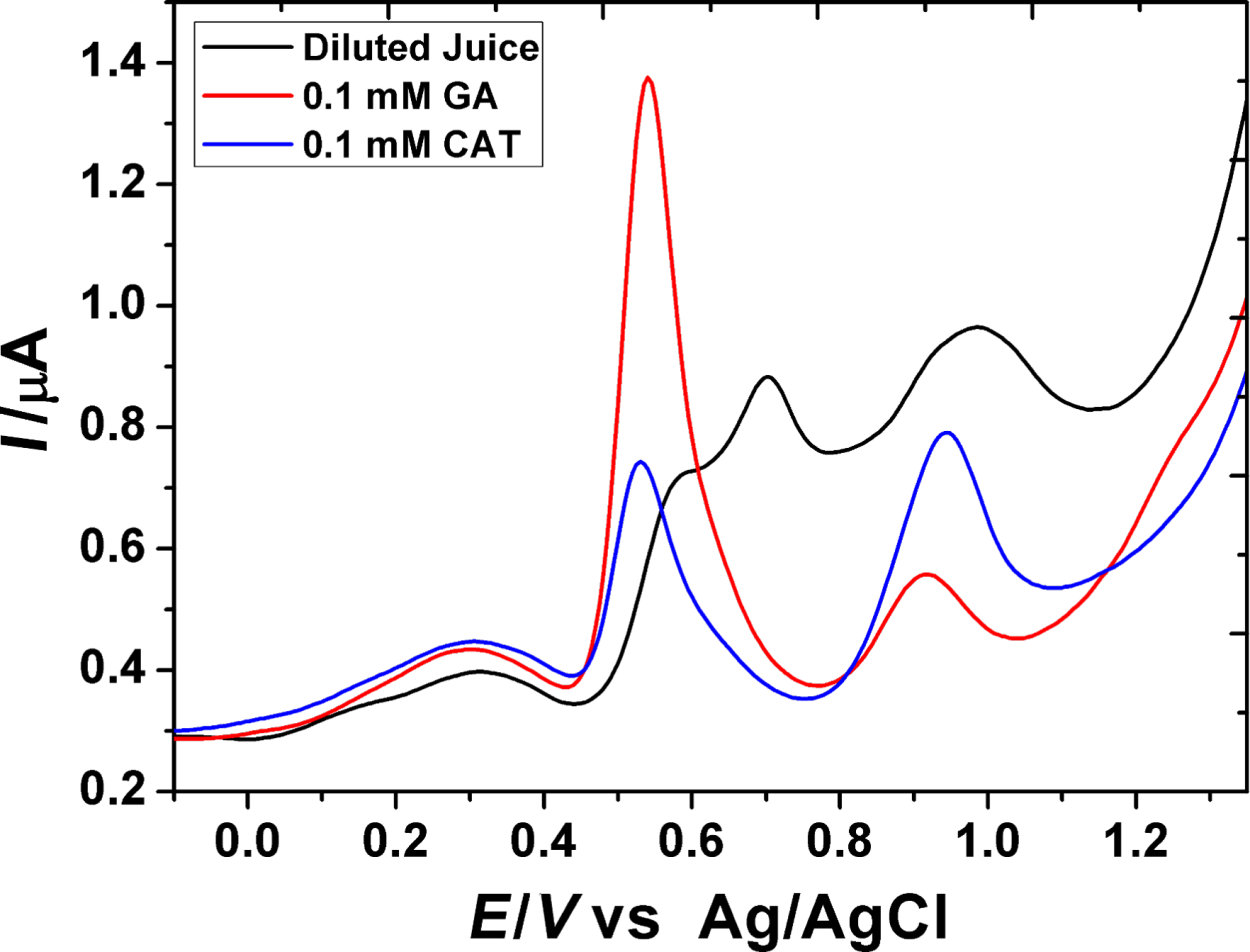
SW voltammograms of a pomegranate juice sample (black), 0.1 mM GA (red) and 0.1 mM CAT (blue) in 0.2 M H_3_PO_4_ (pH 2.0) on the PGA/MWCNT/GC modified electrode.

**Figure 8:**
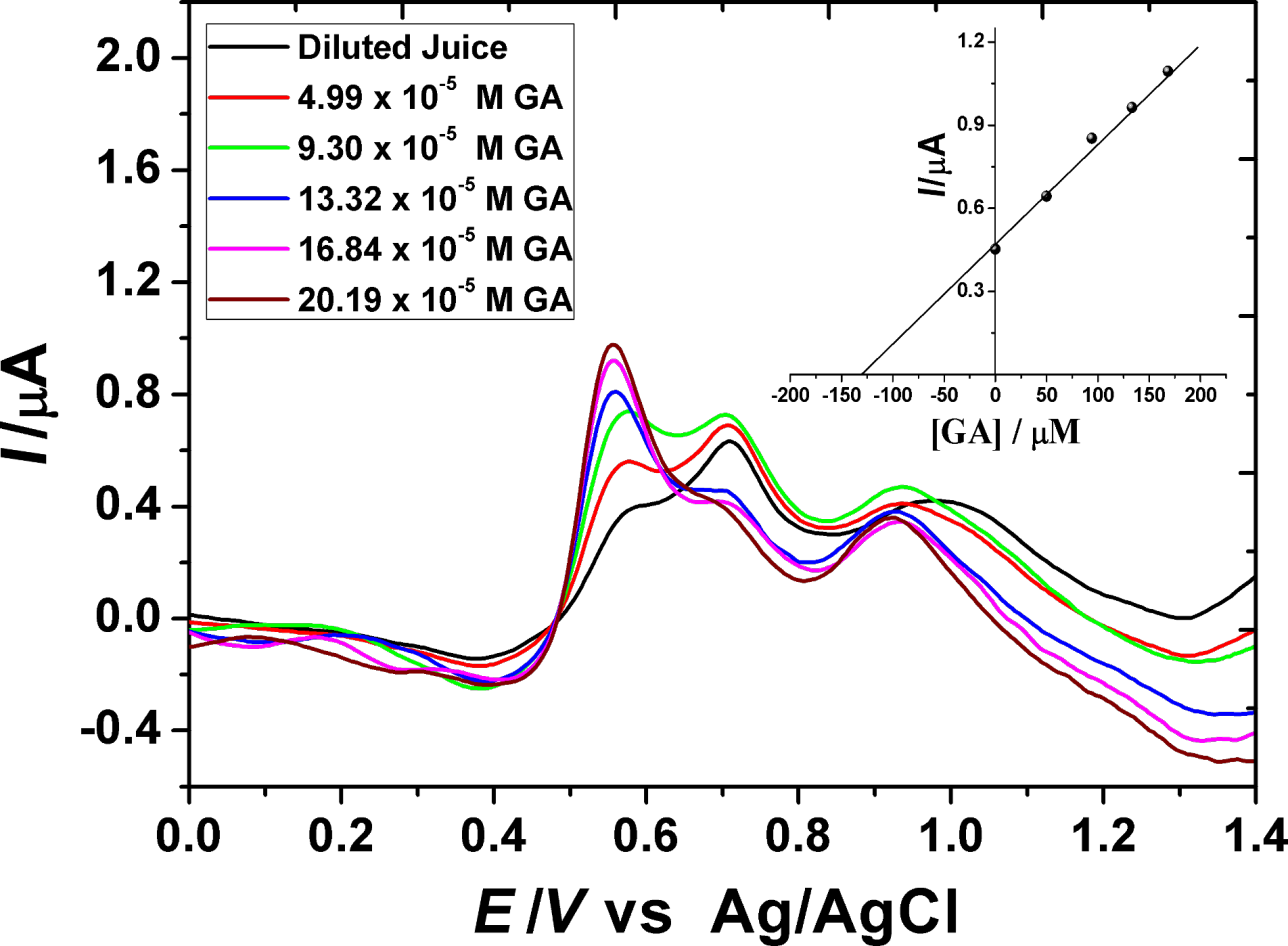
SW voltammograms obtained at optimal conditions of a pomegranate juice sample upon addition of different concentrations of GA for determination of TPC. Inset: the analytical results.

Figure 7 shows the square-wave voltammogram of a fresh pomegranate juice sample (1:10 dilution), 0.1 mM catechin (CAT) and 0.1 mM GA in 0.2 M H_3_PO_4_ (pH 2.0) on PGA/MWCNT/GCE. The voltammogram shows three anodic peak signals at 0.60, 0.70 and 1.0 V. These signals can be attributed to the oxidation of different polyphenolic compounds, including GA and CAT, as judged from a comparison of their voltammetric response. Thus, it can be concluded that the first wave may dominate the response of GA. Gallic acid is one of the most common references for evaluation of the antioxidant total phenolic content of foodstuff. From the peak current value, the total phenolic content (TPC) can be measured. For confirming the validity of the method, the SWAdSV technique was used for the determination of TPC in fresh pomegranate juice sample. The TPC was determined upon using the standard addition method with a standard solution of GA under the same procedure described earlier with the optimal parameters. The successive increase in concentration of the GA solution was added to the juice sample solution. The typical data obtained are given in Figure 8. The TPC is expressed as mg of gallic acid equivalents (GAE) per liter of juice (mg GAE L^−1^). The TPC value is estimated to be 225.0 mg L^−1^. To confirm the electrochemical method for determination of the TPC in pomegranate juice, the colorimetric method was conducted using the same juice sample. The estimated TPC value is found to be 277.4 mg L^−1^. The observed higher value can be attributed to the fact that all phenols in the real samples can be detected by this method, and furthermore, the Folin–Ciocalteu reagent may react with many nonphenolic substances [31]. This problem can be resolved by using the sensor since the nonphenolic compounds have no interference with the response of the polyphenols. In comparison with Folin–Ciocalteu spectrophotometric procedures, the sensor exhibits better results in terms of sensitivity and selectivity.

## Experimental

### Reagents

Gallic acid, potassium hexacyanoferrate, multiwalled carbon nanotubes (MWCNTs), ethanol, nitric acid, and sulfuric acid were purchased from Sigma-Aldrich (France) and used without any further purification. The stock solution of GA (0.01 M) was prepared in double-distilled water. The stock solutions were protected from light, kept in a refrigerator, and used within the same day of preparation. Pure nitrogen was used for degassing the test solution prior to and throughout the electrochemical measurements. Phosphoric acid solution was used as a supporting electrolyte. Double-distilled water was used for preparation of all solutions. Freshly prepared standard solutions of gallic acid were prepared by dilution of the stock solution with 0.2 M phosphoric acid.

### Instrumentation

Cyclic and square wave voltammetric, chronoamperomeric and chronocoulomeric experiments were performed by using an Autolab PGSTAT128N Potentiostat/Galvanostat (Eco-Chemie, Utrecht, The Netherlands) coupled with NOVA 1.10 software. An electrochemical sensor is comprised of three electrodes: the working (bare or modified glassy carbon electrodes), the reference (Ag/AgCl, aqueous KCl, 3.5 M) and the auxiliary Pt wire electrodes. The surface morphology of a (PGA/MWCNT) composite film was examined using a JOEL scanning electron microscope (JSM T200, Japan) with an electron beam energy of 30 kV. For this purpose, a thin layer of gold (50 Å) was deposited using physical vapor deposition. The pH measurements were performed using a bench top pH meter (HI 2210, HANNA Instruments, Romania) with a combined pH reference electrode. The absorbance of the samples was measured using a V-750ST UV–vis Spectrophotometer (JASCO International Co., LTD., Hachioji, Tokyo, Japan) with Spectra Manager 2 software.

### Preparation of multiwalled carbon nanotube (MWCNT) suspension

Firstly, MWCNTs were treated with a mixture of sulfuric acid and nitric acid (3:1 vol.) for 6 h to remove impurities, reduce bundle sizes and to generate functional groups on their surface. This was then washed several times with double-distilled water until the washing was neutral and then dried at about 70 °C as described by Abdel-Hamid et al. [22]. Secondly, a suspension of MWCNTs was prepared by sonicating a mixture of 30 mg of sodium dodecyl sulfate, 5 mg of treated MWCNTs, 1 mL of *N,N-*dimethylformamide and 1 mL of ethanol for 4 h to form a stable black suspension.

### Sensor construction

The glassy carbon electrode surface was polished with 0.05 μm alumina water slurry using a polishing cloth until the electrode surfaces developed a mirror finish. Then, it was rinsed thoroughly with double-distilled water. The PGA/MWCNT/GC modified electrode was prepared as follows. 20 µL of the MWCNT suspension was drop-casted onto the polished clean glassy carbon electrode to prepare a MWCNT/GC electrode and left for 6 h to dry. The PGA/MWCNT/GCE was fabricated by potentiostatic electropolymerization of gallic acid on the MWCNT/GC electrode by applying an anodic potential of 1.0 V vs Ag/AgCl for 60 s. The prepared electrode was washed several times to remove the electrolyte and the monomer. The electrode was then ready for electrochemical use. The bare and the modified GC electrodes were electrochemically cleaned before the measurements using cyclic voltammetry in a potential range between 0.2 and 1.0 V for 10 cycles at a scan rate of 50 mV/s.

### Preparation of pomegranate juice sample and determination of total phenolic content

The pomegranate juice was obtained by peeling the fruits by hand and the seeds were liquefied using a hand press. The obtained juice was filtered off through a Whatman filter paper (No. 1). An aliquot of 10.0 mL of pomegranate juice was transferred to a calibrated flask and diluted to a final volume of 100 mL with double-distilled water (1:10 dilution). An aliquot of 100 μL of the dilution was used for the electrochemical standard addition measurements. The total phenolic content in the pomegranate juice sample obtained from the standard addition method was compared with the spectrophotometric Folin–Ciocalteu result [32]. The Folin–Ciocalteu method is based on the reduction of phosphotungstic acid in an alkaline solution, which yielded the phosphotungstic blue. The absorbance of the formed phosphotungstic blue is relative to the number of aromatic phenolic groups and is used for their quantification, using gallic acid as a standard. An aliquot of 20 μL of the raw juice, 1.58 mL of water and 100 μL of Folin–Ciocalteu reagent was mixed. After waiting 8 min, 300 μL of a solution of sodium carbonate (200 g L^−1^) was added. After mixing, the prepared solution was left 2 h at 20 °C and then the absorbance was determined at 765 nm against the blank. The results were expressed using gallic acid as a standard (mg GAE L^−1^).
